# Sexual Assault and Forensic Exam Offers in the Emergency Department: A Retrospective Study

**DOI:** 10.5811/westjem.48540

**Published:** 2026-01-09

**Authors:** Kirsten Walton, Maria Diaz, Colton Hood, Neal Sikka, Philip Ma, Sonal Batra

**Affiliations:** * George Washington University, Department of Emergency Medicine, Washington, DC; † George Washington University, School of Medicine and Health Science, Washington, DC; ‡ George Washington University, School of Medicine and Health Science, Biomedical Informatics Center, Washington, DC

## Abstract

**Introduction:**

Patients who report sexual assault in the emergency department (ED) have a legal right to a forensic exam. Emergency departments that do not provide such exams must offer transfer to a forensic site. Little is known about the factors influencing whether patients are offered a forensic exam and complete the transfer. In this study we aimed to identify patient characteristics associated with being offered a forensic exam in an ED that does not perform them on site.

**Methods:**

We conducted a retrospective chart review of adult patients presenting to a single, urban, academic ED between January 2017–December 2019. The ED receives over 75,000 visits annually and refers patients to an external site for forensic exams. Using keywords “sexual assault” or “rape” we identified charts that included whether the visit involved an initial report of sexual assault. Charts were abstracted for demographics, insurance status, psychiatric history, clinician concern for acute mental illness or substance use, and mode of arrival. The primary outcome was whether a forensic exam was offered. Statistical analyses included chi-square tests and penalized logistic regression.

**Results:**

Of 167 charts reviewed, 108 met inclusion criteria. Of these, 94 patients (87.0%) were offered a forensic exam and 14 (64.8%) accepted transfer. Patients who were offered exams were younger (mean age 29.9 vs 36.8 years, P = .05), more likely to arrive ambulatory (69.1 vs 42.9%, P = .02), and less likely to have a psychiatric history (31.9 vs 71.4%, P = .01). Clinician concern for acute psychiatric illness or substance use was significantly associated with not offering a forensic exam (64.3 vs 16.0%, P < .001). In regression analysis, this concern was the only independent association of not being offered a forensic exam (adjusted odds ratio 0.16, 95% CI, 0.03–0.76, P = .02). Additionally, 23.1% of patients were uninsured, significantly higher than the local rate of 2.7%.

**Conclusion:**

Patients in the ED who report sexual assault are less likely to be offered a forensic exam if they present with signs of acute mental illness or substance use disorder. These findings highlight the need for standardized protocols and advocacy to ensure equitable access to forensic exams, especially for patients with behavioral health needs or without insurance.

## INTRODUCTION

Sexual violence is a public health crisis in the United States. Contact sexual violence, defined as rape, sexual coercion, forced penetration, and/or unwanted sexual contact, affects 54.3% of adult women and 30.7% of adult men in the US.^1^ These acts of sexual violence have significant acute and chronic repercussions for the victims. In the acute period after sexual assault, 1 in 7 female victims reported sexually transmitted infections, 1 in 3 were injured, 1 in 7 became pregnant, and 2 in 3 were fearful and/or concerned for safety.^1^ Victims of sexual violence also have higher long-term rates of chronic medical conditions and activity limitations.^1^

It is estimated that 21% of victims of sexual violence will seek acute medical care, which results in greater than 55,000 emergency department (ED) visits related to sexual assault in the US each year.^2^,^3^ The ED, therefore, has a unique opportunity to make a significant impact on the acute and long-term care of victims of sexual violence. Federal, state, and local laws regulate ED care for victims of sexual assault. The Survivor’s Bill of Rights Act of 2016 established and updated statutory rights nationwide that include the right to…not be prevented from receiving a forensic medical examination and not be charged for an examination; (2) have a sexual assault evidence collection kit (ie, a rape kit) preserved for 20 years or the maximum applicable statute of limitations, whichever is shorter; (3) receive written notification prior to destruction or disposal of a rape kit; and (4) be informed of these rights and policies.^4^

Therefore, every ED is required to offer or facilitate a free sexual assault forensic exam that includes evidence collection, known commonly as a Physical Evidence Recovery Kit (PERK), to every patient who reports sexual assault.

In addition to the forensic exam and PERK, ED care should provide comprehensive services for key medical and behavioral interventions. The standard of care includes sexually transmitted disease prevention, emergency contraception, injury identification and treatment, and trauma-informed care practices to reduce the acute and long-term psychological impact of sexual violence.^5^ Many EDs also employ a patient advocate who may provide acute crisis intervention at the initial ED visit and/or subsequent long-term advocacy and connection to community resources for the victim.

Previous studies have consistently demonstrated that Sexual Assault Nurse Examiners (SANE) and patient advocates together provide higher rates of comprehensive medical services and proper completion of forensic exams compared to ED care without these resources. The SANEs also have been shown to establish a compassionate attitude, provide clear explanations, and empower individual agency for victims of sexual assault.^6^ In contrast, ED staff in departments that do not have SANEs have been observed to convey skepticism of the victims in 53.2% of visits and to blame them for the circumstances of assault in 28.4% of visits.^6^

Recent surveys have found that only 55.3% of EDs in the US often or always have SANE services.^6^ To compensate for the lack of on-site SANE care, EDs have developed different models of providing the federally mandated forensic services. Some EDs will transfer patients to SANE centers based on the evidence that SANEs provide higher quality comprehensive services.^6^,^7^ Other EDs have adopted telemedicine programs using remote SANE consultation in real time.^8^, ^9^ Finally, some EDs will provide the forensic exams and PERK collection without a SANE.^10^ Little is known about the factors that impact sexual assault victim care in the 44.7% of EDs that transfer patients to forensic sites. Previous studies have examined ED staff comfort and knowledge in caring for victims of sexual assault^11^; however, much less is known about the quality of patient care in systems that transfer patients. We aimed to answer the following question: In an ED that does not provide forensic exam services, what factors affect whether patients reporting sexual assault are offered a forensic exam and are ultimately transferred to a location with a SANE.

**Population Health Research Capsule**

What do we already know about this issue?

*Patients reporting sexual assault have a legal right to forensic exams, but access in emergency departments (ED) without on-site exams is inconsistent*.

What was the research question?

*What factors influence whether ED patients reporting sexual assault are offered a forensic exam?*

What was the major finding of the study?

*Clinician concern for acute mental illness or substance use reduced exam offers (odds ratio .16, 95% CI, 0.03–0.76, P = .02)*.

How does this improve population health?

*Identifying disparities in forensic exam offers supports standardized ED protocols to ensure equitable care for sexual assault survivors*.

## METHODS

### Study Setting

We conducted a retrospective, cohort study from January 1, 2018–December 31, 2022 in a single, urban, academic ED in the US with 75,000 annual visits that does not provide sexual assault forensic exams. The study was deemed exempt by the institutional review board (IRB#NCR224704). No funding was received for this study. During the study period, the ED policies related to patients who reported sexual assault were to offer the patient transfer to a local ED that does provide forensic exams. The acute window for the forensic exam is 120 hours or less from assault, as defined by the local SANE program. Patients that decline transfer are offered a patient advocate on the phone, the opportunity to make a police report, and comprehensive sexually transmitted infection prevention and emergency contraception. Patients may choose any combination or none of these services. We performed a retrospective chart review to evaluate factors that impact whether patients reporting sexual assault in the acute window are offered transfer for a forensic exam. Our study design and the following methodology adheres to the following elements of optimal retrospective chart review: abstractors trained before data collection; definition of inclusion and exclusion criteria for case selection; definition of variables; use of data abstraction forms; measurement and discussion of inter-observer reliability; description of the medical record database; description of the sampling method; description of statistical management of missing data; and IRB approval.^13^

### Patient Selection

Patient charts were selected through a keyword search for “sexual assault” or “rape” mentioned anywhere in the chart. Physician and nursing notes were included in the initial query. We included all ED charts over the three-year period in the initial search. Next, two reviewers read each chart to determine whether the patient encounter met inclusion criteria. Each chart was read by both reviewers. Reviewer discrepancies were resolved through consensus discussions. Reviewers were trained to abstract variables using the study protocol. Charts were abstracted for inclusion criteria, predefined variables, and the study outcomes. For inclusion, the patient had to be ≥ 18 years of age, and the ED encounter must have included the initial report of sexual assault. The variables included the following: age; race; sex; mode of arrival; insurance; location of assault; a history of psychiatric illness; and whether there was clinician concern for psychiatric illness or substance use at the initial encounter for sexual assault in the ED. The primary outcome was whether the emergency clinician offered the patient a sexual assault forensic exam if they presented within the acute window.

### Quantitative Variables

We grouped detailed insurance information into Medicaid, Medicare, private insurance, or uninsured/discount. City- and county-level data, when available, was gathered for the variable “Jurisdiction of Assault”, which were grouped as follows: District of Columbia (DC); Maryland (MD); Virginia (VA); unknown, or other. The remaining variables were not grouped and are largely binary, aside from age, which was kept as a continuous variable. For the purposes of regression, to avoid issues with collinearity, some additional groupings were made. For “mode of arrival,” we combined “brought in by ambulance” and “Advanced Life Support (ALS) ambulance,” as well as “law enforcement” and “other.” For “location of assault,” we grouped locations by DC, MD and VA to account for the perfect separation in outcomes. For the same reason, we decided to code the lone “male to female” participant as female for the purposes of this study. The variable “clinician concern for psychiatric illness or substance use at the initial encounter for sexual assault in the ED” was determined based on two-reviewer agreement on chart review. The variable was defined by whether the patient chart documented clinician concern or description of acute psychosis, delusions, hallucinations, or erratic behavior. We defined this variable as acute mental illness or substance use, as the emergency clinicians of record on chart review were often unable to determine the etiology of behavior during the initial patient encounter. The variable “history of psychiatric illness” was also determined based on two-reviewer agreement on chart review. We included any historical diagnosis of psychiatric illness of any severity. If any variable was not listed in the chart, or missing, it was categorized as “unknown.”

### Statistical Methods

We compared patient characteristics between those who were offered a sexual assault forensic exam (n = 94) and those who were not offered a forensic exam (n = 14), using appropriate statistical tests. Continuous variables (eg, age) were compared using Student *t*-tests, while we compared categorical variables (eg, race, sex, mode of arrival, insurance status, location of assault, psychiatric history, concern for psychosis/substance use at visit) using chi-square tests. A significance level of .05 was used for all statistical tests.

To identify factors independently associated with forensic exam offering while accounting for the small sample size (n = 108) and outcome imbalance, we performed a penalized logistic regression with L1 regularization using the generalized linear model function in the statsmodels Python package (Python Software Foundation, Wilmington, DE). This approach helps prevent overfitting and manages separation issues common in datasets with rare outcomes. The model included the following potential associations: age; race; sex; mode of arrival; insurance status; location of assault; psychiatric history; and concern for psychosis/substance use at visit. Results are presented as adjusted odds ratios with 95% confidence intervals.

## RESULTS

### Exploratory Data Analysis

We identified 167 patient charts in the initial keyword search. A total of 108 patients were included in the analysis. Inter-rater agreement was near perfect, Cohen kappa 0.95. We excluded 59 charts because sexual assault was not reported by the patient, or the ED visit was not the initial encounter for sexual assault. Of the included patients, 94 (87.0%) were offered transfer for sexual assault forensic exam and 14 (13.0%) were not offered the exam, or there was no record of the exam being offered. Table 1 presents the demographic and clinical characteristics of the study population.

Patients who were offered forensic exams were significantly younger than those not offered an exam (mean age 29.9 ± 9.7 vs. 36.8 ± 11.3 years of age, *P* = .05). Mode of arrival differed significantly between groups (*P* = .02), with a higher proportion of patients arriving ambulatory in the sexual assault exam-offered group (69.1 vs 42.9%). Insurance status also showed significant differences (*P* = .02), with patients having private insurance (40.4 vs 21.4%) or being uninsured (25.5 vs 7.1%) more likely to be offered the forensic exam compared to those with Medicaid (29.8 vs 50.0%) or Medicare (4.3 vs 21.4%).

Notably, patients without a psychiatric history were more likely to be offered the forensic exam (68.1 vs 28.6%, *P* = .01). Similarly, patients without concerns for psychosis at the time of visit were more frequently offered the forensic exam (84.0 vs 35.7%, *P* < .001). Among patients offered a transfer for the forensic exam, 74.5% accepted it, while none of the patients in the non-offered group had the opportunity to accept. No significant differences were observed between groups regarding race, sex, or jurisdiction of assault.

### Regression Results

In the penalized logistic regression model adjusting for potential confounders, concern for psychiatric illness or substance use at the time of visit emerged as the only significant independent association of forensic exam being offered (adjusted odds ratio [AOR] 0.16, 95% CI, 0.03–0.76, *P* = .02). Patients with emergency clinician concern for acute mental illness or substance use had 84% lower odds of being offered the forensic exam compared to those without such concerns, after controlling for age, psychiatric history, race, sex, mode of arrival, insurance status, and jurisdiction of assault.

Age showed a trend toward lower odds of forensic exam being offered with increasing age (AOR 0.96, 95% CI, 0.89–1.03, *P* = .27), although this did not reach statistical significance. Similarly, while psychiatric history was associated with lower odds of a forensic exam being offered in unadjusted analysis, this association was attenuated after adjustment for other factors (AOR 0.81, 95% CI, 0.15–4.46, *P* = .80). Other factors, including race, sex, mode of arrival, insurance status, and assault location, did not show significant independent associations with the likelihood of being offered the forensic exam in the adjusted model. Regression results detailed in Table 2.

## DISCUSSION

In this retrospective, cohort study of adult patients reporting sexual assault in the ED, we found that patients were less likely to be offered a SANE forensic exam if the clinician observed behavior consistent with acute mental illness or acute substance use at the initial ED encounter. There were also non-statistically significant but observed trends of lower rates of forensic exam being offered for patients who were older or who had a history of psychiatric illness. In addition, the observed percentage of patients reporting sexual assault who were uninsured (23.1%) was much higher than the general percentage uninsured in the local population (2.7%).^13^

### Acute Mental Illness and Substance Use

Our study found that 40% of patients had a history of psychiatric illness, which is consistent with previous studies demonstrating that 26–47% of patients evaluated for forensic exam by a SANE had a history of psychiatric illness and were more likely to be victims of sexual assault in their lifetime.^14^–^18^ There are numerous case reports of patients with acute mental illness reporting sexual assault being denied a forensic exam; however, there is a lack of research demonstrating acute mental illness as a risk factor.^19^ In our study, 22% of patients reporting sexual assault demonstrated behavior consistent with acute mental illness or acute substance use. Substance use by victims and perpetrators prior to sexual assault is common. Seifert found that 51% of sexual assault victims reported substance use prior to their assault.^20^ Acute psychosis not attributed to psychiatric illness or substance use during an ED visit for sexual assault is also common; sexual assault itself may lead to trauma-induced psychosis.^21^

There are numerous reasons a clinician may not offer a forensic exam to a patient with acute signs of mental illness or substance use. The emergency clinician may be motivated by beneficence and non-maleficence and have concern for the patient’s ability to consent to the exam, concern that the invasive forensic exam will exacerbate acute psychosis or retraumatize the patient, or the clinician may prioritize treating the acute mental illness over the forensic exam. The clinician may also be impacted by personal bias, lack of trust or belief of assault, belief that the report of sexual assault is motivated by secondary gain, or personal experience.^22^

The complex decision to offer a forensic exam to a person demonstrating acute mental illness was explored in depth by Miles et al. In their paper, the authors based their guidance on the American Disabilities Act that prohibits discrimination based on disability defined as, “a physical or mental impairment that substantially limits one or more major life activities or a record of having such an impairment or being perceived by others as having such an impairment.”^23^ Therefore the authors found that, “ if a patient with [mental illness] requests treatment for sexual assault, there must be a high bar for denial of that request.”^22^ Patients who request a sexual assault forensic exam, are cooperative, and demonstrate capacity should always be offered SANE services regardless of behavior consistent with acute mental illness or substance use.^22^

In our study there was no documentation of capacity evaluations for patients presenting with behaviors consistent with acute mental illness or substance use. Furthermore, there was no documentation that the decision not to offer a SANE examination was attributed to clinician concern regarding a lack of capacity. In the absence of clearly documented capacity assessments and serial evaluations for changes in mental status during the ED encounter, it remains imperative that all cooperative patients who demonstrate capacity are offered a SANE examination, irrespective of co-occurring mental illness or substance use.

Our study demonstrates that there is a continued need to advocate for patients with acute mental illness to receive equitable access to sexual assault forensic exams and comprehensive sexual assault services. Through research, education, and advocacy, emergency clinicians can improve sexual assault care for this vulnerable patient population. In addition, further work should establish clear and standardized guidelines for emergency clinicians on when to offer the SANE forensic exam for patients with acute mental illness. Standardized guidelines could limit clinician bias and reduce the ethical dilemma of when to offer forensic exams.

### Health Insurance

In our study, a higher proportion of patients reporting sexual assault were uninsured (23.1%) or enrolled in Medicaid (32.4%) compared to the local population (2.7% and 24%, respectively).^13^,^24^ These findings are consistent with previous studies, which have reported that up to 29.5% of patients reporting sexual assault were covered by Medicaid and 27.7% were uninsured or self-pay.^2^ However, limited research has compared rates of uninsurance among patients reporting sexual assault to those in the general population. Further investigation is needed to understand the underlying factors contributing to higher rates of uninsurance in this population. This is particularly important because, although forensic services are provided free of charge under the Victims of Crime Act through the Crime Victims Fund, patients may still perceive these services as costly—potentially deterring them from seeking care.^25^ A better understanding of the relationship between sexual assault and lack of insurance could help clinicians more effectively support and advocate for patients, including assisting them with health insurance enrollment.

## LIMITATIONS

The study was limited by the number of patients included in the sample size. The relatively small sample size reduces the statistical power of the results, creates wider confidence intervals due to the greater impact of outliers, and limits the generalizability of the results. In addition, charts may have been missed for inclusion if the patient reported sexual assault, but the documentation included alternate descriptors other than the keywords “sexual assault” or “rape.” The likelihood of missing data is possible due to the broad range of terms used to describe sexual assault. The study was also limited by the inherent nature of retrospective chart review: The variables collected were not standardized at the time of chart documentation. Therefore, there may be missing data, categorized as “unknown,” if clinicians did not elect to document specific variables.

The external validity is limited as the study was conducted at a single, academic, urban ED that does not perform sexual assault forensic exams. The EDs that include SANE services on site may have less variance in forensic exam offering as there are fewer barriers to completing the exam. Further research that is multicenter with a larger sample size is needed to expand the generalizability of the results.

## CONCLUSION

Patients presenting to the ED reporting sexual assault are less likely to be offered a forensic exam if the clinician has concerns for acute mental illness or acute substance use. In addition, patients reporting sexual assault are uninsured at a higher percentage compared to the local population. These results suggest that emergency clinicians have the opportunity to provide equitable access to sexual assault forensic exams and to assist victims of sexual assault with health insurance enrollment.

## Supplementary Information



## Figures and Tables

**Table 1:** Demographic and clinical characteristics of patients reporting sexual assault in the emergency department with data stratified by whether patients were offered a forensic exam.

	Overall	Forensic exam not offered	Forensic exam offered	P-value
Number	108	14	94	
Age, mean (SD)	30.8 (10.2)	36.8 (11.3)	29.9 (9.7)	.05
Race, n (%)				
Black	54 (50.0)	10 (71.4)	44 (46.8)	.22
Other/Unknown	17 (15.7)	1 (7.1)	16 (17.0)	
White	37 (34.3)	3 (21.4)	34 (36.2)	
Sex, n (%)				
Female	92 (85.2)	11 (78.6)	81 (86.2)	.64
Male	15 (13.9)	3 (21.4)	12 (12.8)	
Male to female	1 (0.9)	0 (0.0)	1 (1.1)	
Mode of arrival, n (%)				
ALS ambulance	1 (0.9)	0 (0.0)	1 (1.1)	.02
Ambulatory	71 (65.7)	6 (42.9)	65 (69.1)	
BLS ambulance	33 (30.6)	6 (42.9)	27 (28.7)	
Law enforcement	2 (1.9)	1 (7.1)	1 (1.1)	
Other	1 (0.9)	1 (7.1)	0 (0.0)	
Insurance, n (%)				
Medicaid	35 (32.4)	7 (50.0)	28 (29.8)	.02
Medicare	7 (6.5)	3 (21.4)	4 (4.3)	
Private	41 (38.0)	3 (21.4)	38 (40.4)	
Uninsured/discount	25 (23.1)	1 (7.1)	24 (25.5)	
Jurisdiction of assault, n (%)				
District of Columbia	27 (25.0)	0 (0.0)	27 (28.7)	.11
Maryland	9 (8.3)	2 (14.3)	7 (7.4)	
Other	4 (3.7)	1 (7.1)	3 (3.2)	
Unknown	61 (56.5)	11 (78.6)	50 (53.2)	
Virginia	7 (6.5)	0 (0.0)	7 (7.4)	
History of psychiatric illness, n (%)				
No	68 (63.0)	4 (28.6)	64 (68.1)	.01
Yes	40 (37.0)	10 (71.4)	30 (31.9)	
Clinician concern for acute psychiatric illness or substance use at initial encounter, n (%)				
No	84 (77.8)	5 (35.7)	79 (84.0)	< .001
Yes	24 (22.2)	9 (64.3)	15 (16.0)	
Accepted transfer for forensic exam, n (%)				
No	34 (31.5)	11 (78.6)	23 (24.5)	< .001
Yes	70 (64.8)	0 (0.0)	70 (74.5)	
Eloped	4 (3.7)	3 (21.4)	1 (1.1)	

**Table 2:** Logistic regression analysis of factors independently associated with patient being offered a sexual assault forensic exam in a study of 108 visits for sexual assault to the emergency department.

Variable	Odds Ratio	95% Confidence Interval	P-value
Age	0.96	0.89 – 1.03	.24
Clinician concern for acute psychiatric illness or substance use at initial encounter	0.16	0.03 – 0.76	.02
Race			
Other/Unknown vs. Black	1.82	0.16 – 21.02	.63
White vs. Black	1.72	0.22 – 13.16	.60
Sex (Male vs Female)	0.70	0.11 – 4.49	.71
Mode of Arrival			
Ambulance vs. ambulatory	2.29	0.26 – 20.06	.46
Other/law enforcement vs. ambulatory	0.43	0.01 – 14.64	.64
Insurance			
Medicaid vs. private	0.71	0.10 – 5.31	.74
Medicare vs. private	0.61	0.05 – 6.92	.69
Uninsured discount vs. private	2.72	0.21 – 35.86	.45
Jurisdiction of assault (District of Columbia/Virginia/Maryland vs. other/unknown)	2.656	0.41 – 17.17	.31
History of psychiatric illness	0.81	0.15 – 4.46	.80
